# Differentiating Individual Characteristics Associated with Suicidal Ideations, Plans, and Attempts among low-Income Veterans

**DOI:** 10.1177/24705470251348749

**Published:** 2025-06-03

**Authors:** Jack Tsai, Jie Liang, Vahed Maroufy

**Affiliations:** 1 U.S. Department of Veterans Affairs, National Center on Homelessness among Veterans, Washington, DC, USA; 2 49219 School of Public Health, 12340 University of Texas Health Science Center at Houston, Houston, TX, USA

**Keywords:** suicide, veterans, socioeconomic disparities, low-Income, poverty, mental health

## Abstract

**Background:**

Low-income veterans are a group that are at high risk for suicidal behaviors and require clinical attention and research.

**Methods:**

This brief report analyzed data from a nationally representative sample of 985 low-income veterans participating in the National Veteran Homeless and Other Poverty Experiences (NV-HOPE) study in 2021. The lifetime prevalence and correlates of three levels of suicidal behaviors were analyzed, including suicidal ideation (SI), having a suicidal plan (SP), and making a suicide attempt (SA).

**Results:**

In the sample, 17.6% reported any SI, 7.0% reported any SP, and 4.5% reported any SA. Multivariable analyses revealed that compared to veterans who only reported SI, those who reported SP had overall lower mental health functioning scores (aOR = 0.97, 95% CI = 0.95–0.99). Compared to veterans who reported only SP, those who reported SA were two times more likely to be unmarried (aOR = 2.38, 95% = 1.09–5.30).

**Conclusion:**

These findings suggest a few factors may be driving differences between veterans who engage in different levels of suicidal behaviors, and these factors may be important treatment targets.

## Introduction

Suicide is a leading cause of death in the United States.^1^ While suicidal ideation (SI) is relatively common in the general population with an estimated lifetime prevalence of about 8.4–13.5%,^2,3^ the prevalence of individuals having a suicidal plan (SP) is less common with an estimated lifetime prevalence of about 3.9%.^3^ A suicide attempt (SA) is even less common from an epidemiological perspective with an estimated lifetime prevalence of 2.4–4.6%.^2,3^ A review of cross-national studies across 17 countries found the lifetime prevalence of SI, SP plans, and SA is 9.2%, 3.1%, and 2.7%, respectively.^4^ Many studies have examined individual characteristics associated with SI, SA, and SP as phenomena.^5,6^ However there have been fewer studies that have compared individuals with different levels of suicidal behaviors such as differentiating between those who had SI but no SA, from those who had SA but no SP.

Suicide is the top clinical priority of the United States Department of Veterans Affairs (VA) and there is great concern about the well-being of military veterans.^7,8^ Annual analyses as reported in the VA's National Veteran Suicide Prevention Annual Report have revealed veteran suicide rates have not dramatically changed for the past decade, homeless and criminal justice-involved veterans may be at elevated risk, and that a large proportion of veteran suicides occur among veterans not engaged in VA healthcare.^9^ Low-income veterans may be particularly at risk for suicide and other adverse health outcomes.^10,11^ For those reasons, it may be informative to study community samples of low-income veterans to obtain a comprehensive picture of factors associated with veterans’ suicidal behaviors.

To contribute to needed knowledge in this area, this brief report had two aims: 1) to describe the prevalence of any lifetime SI, SP, and SA among low-income veterans; and 2) to compare the characteristics of low-income veterans who have any history of SI, SP, and SA.

## Methods

### Study Sample

Data for this study were drawn from the National Veteran Homeless and Other Poverty Experiences (NV-HOPE) study, which conducted a series of nationally representative surveys of low-income veterans to understand their housing, health, and social needs over time. Although other studies have analyzed NV-HOPE data,^12,13^ the current study analyzed suicide-related variables in the NV-HOPE data that haven’t been reported before. Data were collected through Ipsos and their KnowledgePanel^©^, which manages the largest online research panel of more than 60,000 U.S. households. Panel members are recruited through probability-based sampling, and households are provided with access to the Internet and hardware if needed. To ensure the results represent the target U.S. veteran population, poststratification weights were created using an iterative proportional fitting (raking) procedure based on age, sex, race/ethnicity, education, census region, household income, and metropolitan area data of veterans living in households with incomes below 300% of the federal poverty level, as reported in the 2019 American Community Survey. All participants provided informed consent; the study protocol followed institutional security and human subject procedures by Ipsos; and a de-identified dataset was provided to the authors and thus was deemed exempt by the institutional review board at VA Connecticut Healthcare System. Adult participants were eligible for NV-HOPE study if they were: 18 years or older, served on active duty in the U.S. Armed Forces, and lived in a household under 300% of the U.S. federal poverty level in 2021 (eg, $12,800 for a single individual and $26,500 for family of 4). The baseline survey for NV-HOPE was conducted from October 28, 2021, to December 3, 2021. A total of 1004 veterans were enrolled in this study, but 19 veterans were removed due to missing outcome data, resulting in a study sample of 985 veterans.

### Outcome Variable

The main outcome of this study was suicidal behaviors, including SI, SP, and SA. Veterans were asked to respond to a question about any suicidal behaviors that asked: “Have you ever thought about or attempted to kill yourself?” Six mutually exclusive response options were provided: 1) Never; 2) It was just a brief passing thought; 3) I have had a plan at least once to kill myself but did not try to do it; 4) I have had a plan at least once to kill myself and really wanted to die; 5) I have attempted to kill myself but did not want to die; and 6) I have attempted to kill myself, and really hoped to die. Responses were grouped into four mutually exclusive categories: No suicidal behavior (response 1), SI (response 2), SP (response 3 or 4), and SA (choice 5 or 6).

### Independent Variables

Information on individual sociodemographic, military, and clinical characteristics was collected through self-report. The current housing situation was classified into 3 categories: own house/apartment (with or without a housing voucher); renting/sharing house/apartment; and unstably housed (halfway house, transitional housing, single-room occupancy, or homeless). Current employment status was classified into 4 categories: Employed (full-time/part-time); Unemployed; Retired/disabled; self-employed/others.

For clinical characteristics, any lifetime PTSD was assessed using the PTSD Checklist for DSM-5 (PCL-5)^14^ with a score of 33 or greater coded as a positive screen for PTSD.^15^ The Generalized Anxiety Disorder-2 (GAD-2)^16^ and the Patient Health Questionnaire-2 (PHQ-2)^17^ were used to screen for GAD and major depression, respectively, with scores of 3 or greater on either scale coded as a positive screen. Any alcohol use problems in the past year were assessed with the Alcohol Use Disorder Identification Test-Consumption (AUDIT-C)^18^ with a score of 8 or greater coded as a positive screen. Drug use disorders in the past year were assessed with the 11-item Drug Use Disorder Checklist which was based on the diagnostic criterion outlined in the Diagnostic and Statistical Manual of Mental Disorder (fifth Edition, DSM-5)^19^ with the endorsement of symptoms meeting DSM-5 criteria for drug use disorder as indicative of a positive screen. Overall current physical and mental health functioning were assessed with the Short Form-8 health survey (SF-8).^20^

### Data Analysis

After descriptive analyses were conducted, chi-square tests and analysis of variance were conducted to compare outcomes by individual characteristics. Then, multivariable logistic regressions were conducted to identify individual characteristics significantly associated with the outcome at two levels. At the first level, participants in the SI and SP groups were compared. At the second level, participants in the SP and SA groups were compared. Only variables found in bivariate analyses at each level to be significant at the p < .05 level were included in each respective logistic regression. Specifically, for the comparison between individuals reporting SI versus those reporting SP, the following variables were identified as statistically significant in the bivariate analyses and subsequently included as covariates in the logistic regression model: sex, combat exposure, post-traumatic stress disorder (PTSD), drug use disorder, any lifetime history of receiving treatment for mental health or substance use, and the SF-8 Mental Health Summary Component score.

For the comparison between individuals reporting SP and those who had engaged in a SA, a different set of variables emerged as significant in the bivariate analyses. The following were included as covariates in that logistic regression model: marital status, history of incarceration, positive screens on the GAD-2, drug use disorder, any lifetime history of receiving treatment for mental health or substance use, and reported availability of someone to talk to for mental health support. Adjusted odds ratios (aORs) with 95% confidence intervals (CIs) were calculated. Power calculations revealed the first level analysis had 99.9% power and the second level analysis had 76.6% power. Poststratification weights were applied in all analyses.

## Results

In the study sample, 17.56% reported any SI, 7.01% reported any SP, and 4.47% reported any SA, and 70.96% had no SI/SP/SA. Table 1 shows sociodemographic, military, and clinical characteristics of mutually exclusive groups of veterans with any lifetime SI, SP, SA, and no SI/SP/SA. Compared to the other groups, the SA group was least likely to be married, had the lowest income levels, and was most likely to screen positive for PTSD, depression, GAD, and drug use problems. The SA group was also most likely to have received mental health treatment; and had lower SF-8 Physical and Mental Health Component Summary scores than the other groups.

**Table 1.** Comparison of Individual Characteristics Between Veterans with Different Levels of Lifetime Suicidal Behaviors.

Variables	None	Suicidal ideation	Suicidal plan	Suicidal attempt	Weighted P-value
N(%)	699(70.96%)	173(17.56%)	69(7.01%)	44(4.47%)	<0.001
Male (%)	628 (90.8)	146 (85.4)	50 (74.6)	34 (79.1)	0.01389
Married (%)	408 (58.4)	86 (49.7)	34 (49.3)	12 (27.3)	<0.001
Annual Income (%)					0.006
<=29,999	293 (41.9)	73 (42.2)	26 (37.7)	24 (54.5)	
30,000–49,999	113 (16.2)	30 (17.3)	16 (23.2)	5 (11.4)	
50,000–124,999	293 (41.9)	70 (40.5)	27 (39.1)	15 (34.1)	
Total personal debt(%)					0.1027
<=5000	417 (59.7)	91 (52.6)	34 (49.3)	21 (47.7)	
5001–35,000	135 (19.3)	43 (24.9)	15 (21.7)	10 (22.7)	
35,001–200,000	130 (18.6)	32 (18.5)	15 (21.7)	11 (25.0)	
>200,000	17 (2.4)	7 (4.0)	5 (7.2)	2 (4.5)	
Employed (%)	106 (15.2)	30 (17.3)	18 (26.1)	9 (20.5)	0.01
History of inaccurate (%)	24 (3.4)	9 (5.2)	6 (8.7)	5 (11.4)	<0.001
Combat exposure (%)	202 (28.9)	47 (27.2)	28 (40.6)	15 (34.1)	0.09
Screen positive PTSD (%)	61 (8.7)	38 (22.0)	27 (39.1)	21 (47.7)	<0.001
Screen positive AUD (%)	57 (8.2)	23 (13.3)	12 (17.4)	7 (15.9)	0.007
Screen positive depression (%)	42 (6.0)	34 (19.7)	21 (30.4)	18 (40.9)	<0.001
Screen positive GAD (%)	61 (8.7)	29 (16.8)	17 (24.6)	17 (38.6)	<0.001
Screen positive DUD (%)	12 (1.7)	3 (1.7)	6 (8.7)	5 (11.4)	<0.001
SF8-PCS*	45.44 (11.36)	41.26 (13.10)	40.02 (14.33)	39.70 (15.35)	<0.001
SF8-MCS*	55.79 (9.73)	49.51 (12.82)	45.69 (15.85)	42.57 (16.40)	<0.001
Household size (%)					0.003
1 people	214 (32.4)	56 (33.9)	19 (30.2)	18 (42.9)	
2 people	335 (50.7)	69 (41.8)	25 (39.7)	15 (35.7)	
more than 3 people	112 (16.9)	40 (24.2)	19 (30.2)	9 (21.4)	
Current household					<0.001
Own place	644 (92.1)	158 (91.3)	58 (84.1)	33 (75.0)	
Renting/sharing place	49 (7.0)	13 (7.5)	10 (14.5)	10 (22.7)	
Unstably housed	6 (0.9)	2 (1.2)	1 (1.4)	1 (2.3)	
Emotional Support (%)	444 (63.5)	83 (48.0)	29 (42.0)	15 (34.1)	<0.001
Positive social interaction (%)	371 (53.1)	64 (37.0)	26 (37.7)	13 (29.5)	<0.001
Tangible Support (%)	386 (55.2)	67 (38.7)	27 (39.1)	15 (34.1)	<0.001
Information Support (%)	365 (52.2)	58 (33.5)	25 (36.2)	13 (29.5)	<0.001
Affectionate Support (%)	461 (66.0)	73 (42.2)	31 (44.9)	19 (43.2)	<0.001

Note: The “None” category refers to veterans who reported no suicidal ideation, plan, or attempt. PTSD = Posttraumatic stress disorder, AUD = Alcohol use disorder, GAD = Generalized anxiety disorder, DUD = Drug use disorder, SF-8 PCS and SF-8 MCS = Short-Form Health Survey Physical Component Summary and Mental Health Component Summary scores. The groups shown are mutually exclusive. Values shown are mean (standard deviation).

The first multivariable regression comparing SI and SP groups found a higher Mental Health Component Summary score was associated with a lower likelihood of SP than SI (aOR = 0.97, 95% CI = 0.95-0.99). Although not statistically significant (p = .07), veterans who reported combat exposure were nearly two times more likely to report SP than those who didn’t (aOR = 1.79, 95% CI = 0.94, 3.14).

The second multivariable regression comparing SP and SA groups found that married veterans were less likely to report SA compared to unmarried veterans (aOR = 0.42, 95% CI = 0.19-0.92; or the reverse aOR = 2.38, 95% = 1.09-5.30).

## Discussion

This brief report provides estimates of the lifetime prevalence of any SI, SP, and SA in the low-income U.S. veteran population. About 18% of veterans reported any SI, 7% reported any SP, and 4% reported any SA. These estimates of suicidal behaviors are lower than those reported by a systematic review that found 74% of homeless veterans report any lifetime SI and 15–47% report any lifetime SA^21^; but higher than those reported in the general veteran population, such as 24% and 29% of any lifetime SI among male and female veterans, respectively; and 6% and 12% of any lifetime SA among male and female veterans, respectively.^22^

Our bivariate analyses revealed veterans who reported any SA were different from veterans who reported only SI and veterans who reported only SP, in that they were most likely to be unmarried, to have the lowest income, and to screen positive for various mental health and substance use disorders. This finding is consistent with previous studies that have found lower socioeconomic status, lower social support, and poorer mental health to be associated with SA.^23–25^ It may be important to consider that these veteran characteristics are known to be associated with various other negative psychosocial and health outcomes, including homelessness, criminal justice involvement, and all-cause mortality.^26–28^ Thus, veterans with these characteristics may be at overall greater risk for negative outcomes that includes, but is not specific, to SA.

Interestingly, multivariable analyses found only a few significant factors that differentiated veterans with different levels of suicidal behaviors. Specifically, the only significant factor between veterans who reported SP and those who reported only SI was that veterans with SP had poorer overall mental health functioning, suggesting SP may be mostly driven by the severity of their general mental health. This finding is consistent with studies that have found single risk factors cannot be used to accurately predict suicide-related outcomes and that there are many similar risk factors for a range of suicide-related outcomes.^23^ Among veterans who reported SA versus only SP, the only significant difference was that veterans who reported SA were 2 times more likely to be unmarried, suggesting the importance of marital and social support and as potential targets for intervention to prevent SA among veterans. While social support has widely been recognized as importing in suicide risk among veterans and non-veterans,^29^ there have been few strong evidence-based interventions to target this domain.^30^ Together, our findings indicate the importance of identifying these known risk factors for suicidal behaviors and possibly the few ways in which veterans with different levels of suicidal behaviors might differ in directing suicide prevention interventions.

This study was limited by its cross-sectional data and the small cells of veterans who reported SP and SA in analyses. Our multivariable analyses included some of the key variables, but there may be unmeasured variables that confound the findings. Lastly, our sample focused on low-income veterans and there is unknown generalizability of the findings to the broad veteran population or other adults.

## Conclusions

Study limitations notwithstanding, these findings make an incremental contribution to the literature in understanding how suicidal behaviors and their correlates differ among low-income veterans, a group at high risk for suicide and psychosocial needs. Assessments of suicide risk and treatment plans should plan for different factors associated with these different levels of suicidal behaviors.
